# Direct Modification of Microcrystalline Cellulose with Ethylenediamine for Use as Adsorbent for Removal Amitriptyline Drug from Environment

**DOI:** 10.3390/molecules22112039

**Published:** 2017-11-22

**Authors:** Roosevelt D. S. Bezerra, Régis C. Leal, Mateus S. da Silva, Alan I. S. Morais, Thiago H. C. Marques, Josy A. Osajima, Andréia B. Meneguin, Hernane da S. Barud, Edson C. da Silva Filho

**Affiliations:** 1 Federal Institute of Education, Science and Technology of Piauí, Teresina-Central Campus, IFPI, Teresina 64000-040, PI, Brazil; rooseveltdsb@ifpi.edu.br (R.D.S.B.); thiagohenriquemarques@gmail.com (T.H.C.M.); 2 Federal Institute of Education, Science and Technology of Rio Grande do Norte, Nova Cruz Campus, IFRN, Nova Cruz 59215-000, RN, Brazil; regis.casimiro@ifrn.edu.br; 3 Institute of Chemistry, State University of Campinas, UNICAMP, Barão Geraldo, Campinas 13083-970, SP, Brazil; 4 Interdisciplinary Laboratory for Advanced Materials-LIMAV, UFPI, Teresina 64049-550, PI, Brazil; mateusufpi@gmail.com (M.S.d.S.); alanicaro@gmail.com (A.I.S.M.); josyosajima@ufpi.edu.br (J.A.O.); abagliottim@hotmail.com (A.B.M.); 5 Laboratory of Polymers and Biomaterials (BioPolMat), UNIARA, Araraquara 14801-340, SP, Brazil; hernane.barud@gmail.com

**Keywords:** modified cellulose, amitriptyline, adsorption

## Abstract

Cellulose derivatives have been widely used as adsorbents for the removal of micropollutants such as drugs, dyes, and metals, due to their abundance, low cost and non-contaminating nature. In this context, several studies have been performed searching for new adsorbents (cellulose derivatives) efficient at contaminant removal from aqueous solutions. Thus, a new adsorbent was synthesized by chemical modification of cellulose with ethylenediamine in the absence of solvent and applied to the adsorption of amitriptyline (AMI) in aqueous solution. The modification reaction was confirmed by X-ray Diffraction (XRD), elemental analysis, Fourier Transform Infrared Spectroscopy (FTIR), Thermogravimetry/Differential Scanning Calorimeter (TG/DSC), solid state Nuclear Magnetic Resonance of ^1^H and ^13^C (^1^H-NMR and ^13^C-NMR). Moreover, the effectiveness of reaction was confirmed by computational calculations using Density Functional Theory (DFT) at level B3LYP/6-31G(d). This adsorption process was influenced by pH, time, concentration, temperature and did not show significant changes due to the ionic strength variation. Through these experiments, it was observed that the maximum adsorption capacity of AMI by CN polymer at 298 K, 300 min, and pH 7 was 87.66 ± 0.60 mg·g^−1^.

## 1. Introduction

Several studies have demonstrated the existence of contamination by pharmaceuticals in surface waters and ground waters [1]. These pharmaceuticals are found in water bodies at extremely low concentrations (ng·L^−1^ to μg·L^−1^), however, they may be persistent in the environment and their accumulation may be toxic or be irreversible in a biological system [2]. Examples of drugs found in water bodies are psychiatric ones, such as anxiolytics, sedatives and antidepressants. These medications are among the most prescribed around the world, and consequently, have been frequently found in water bodies [3].

**Figure 1:**
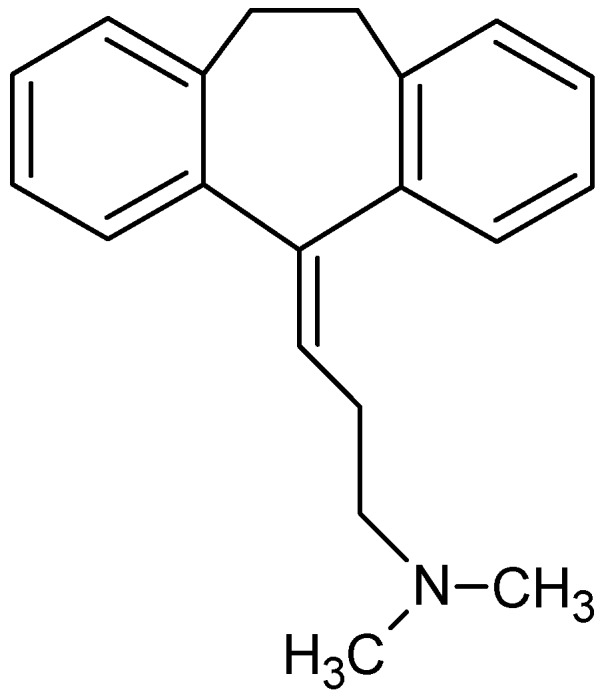
Molecular structure of amitriptyline (AMI).

A tricyclic antidepressant widely used and one of the oldest in the market is the amitriptyline (AMI, Figure 1). Although AMI is comparatively more toxic (especially at low doses) than the selective serotonin reuptake inhibitors (SSRI), it is still widely prescribed due to its low price [1]. The extensive use of AMI has resulted in its frequent detection in wastewater, surface runoff and effluent from sewage treatment plants, and as such, these waters could potentially reach agricultural land through the application of municipal biosolids or reclaimed water use [4]. Therefore, many techniques for the removal of this contaminant from aqueous media have been studied, such as: advanced oxidation processes (UV radiation and Fenton reagent) [5], membrane bioreactors [6], distillation membranes [7], and adsorption [2,3,4], among other techniques.

Among the mentioned previously techniques, adsorption is an important method that offers greater flexibility, beyond the possibility that the treated effluent can be reused. Further, adsorption is often a reversible process, and regeneration of the adsorbent is possible, which allows a good operational economy [8]. Different materials are used as adsorbents, such as activated carbon, chitosan, cellulose, silicates, phyllosilicates and others. Usually, adsorbents are chemically modified in order to increase their intrinsic adsorption capacity [9].

Cellulose is widely explored as an adsorbent because it has a surface susceptible to be chemically modified, which promotes the increase of its capacity of adsorption of contaminants from aqueous media. On this topic, several surface modification reactions of cellulose are studied in which we can highlight the incorporation of alkaline centers on cellulose surface from of nitrogen-containing compounds. The incorporation of this alkaline center, in the cellulose structure, can occur through a variety of chemical reactions, using various nitrogen precursors, such as aminoethanethiol [8], ethylenediamine [10], 2-aminomethylpyridine [11] and 1,4-diaminobutane [12]. Studies showed, in aqueous medium, an interaction between the nitrogen incorporated to cellulose and contaminants (metals and dyes), in a heterogeneous system using the solid/liquid interface [13]. For example, cellulose modified by ethylenediamine proved efficient in the adsorption of divalent cations (Cu^2+^, Ni^2+^ and Zn^2+^) [14], as the cellulose modified by ethylenediamine, with an oxidized cellulose derivative as intermediate, was efficient in the removal of acid red GP, congo red 4BS and reactive light-yellow K-4G anionic dyes [15].

Furthermore, several studies have shown the application of modified cellulose in the removal of several different drugs. For example, cationic and anionic forms of cellulose were synthesized by introducing quaternary ammonium groups and carboxyl groups and were efficient in the adsorption of the ciprofloxacin from aqueous medium [16]. The cellulose modified by grafting a quaternary ammonium salt was an effective adsorbent or the removal of amoxicillin from aqueous solution [17]. The cellulose phosphate obtained by a reaction between cellulose and sodium trimetaphosphate (STMP) at pH 10 was applied in the removal of the pharmaceutical drug acetaminophen from aqueous media having a good adsorption against this drug [18].

Although there are no reports of the use of nitrogenous cellulose derivatives for the adsorption of drugs from aqueous media, the literature shows, for example, that adsorbents produced by pyrolysis of paper mill sludge were efficient in removal of the contaminant citalopram from water [19]. Another study showed that adsorbent materials produced by chemical activation of primary sludge (PS) from a paper mill industry were effective at adsorbing fluoxetine from water [20]. In relation to the drug amitriptyline, studies show that the adsorbents more used are activated carbons, Ca-montmotillonite (SAz-2), and kaolinite [1,2,4], evidencing once again the need to show the efficiency of nitrogenous cellulose derivatives in removal of the drugs from aqueous medium, such as amitriptyline, fluoxetine, citalopram, among others.

Therefore, it is necessary to conduct studies in nitrogenous cellulose derivatives and its application in drug removal. Therefore, this study aimed at the chemical modification of cellulose by ethylenediamine in the absence of solvent, to characterize the product by X-ray diffraction (XRD), elemental analysis, Fourier transformed infrared spectroscopy (FTIR), thermogravimetry/differential scanning calorimetry (TG/DSC), solid state (^1^H-NMR and ^13^C-NMR), and apply it in adsorption experiments (removal), varying time, pH, concentration, temperature and ionic strength. Finally, the experimental data were adjusted to different physico-chemical models of kinetics, isotherms and thermodynamic. Moreover, computations were performed (Density Functional Theory (DFT), the QST3-Synchronous Transit-Guided Quasi-Newton (STQN, *N* = 3) and the Intrinsic Reaction Coordinate (IRC) methods) for determining the reaction mechanism and confirmation of products generated during the reaction between cellulose and ethylenediamine.

## 2. Results and Discussion

The studies reported in the literature about chemical modification of the cellulose surface by ethylenediamine showed this reaction occurs easily when cellulose is firstly chlorinated (halogenation reaction) and subsequently reacts with ethylenediamine. This intermediate step is used to increase the cellulose reactivity, and therefore, increase the amount of ethylenediamine incorporated on the cellulose [21]. However, cellulose halogenation, besides being an expensive reaction, from an economical perspective, it also promotes the formation of a toxic intermediate, and may generate co-products hazardous to the environment. Thus, in this work, cellulose was reacted directly with ethylenediamine in the absence of solvent, without the formation of any intermediate, in other words, without the halogenation step.

### 2.1. Product Characterization

**Figure 2:**
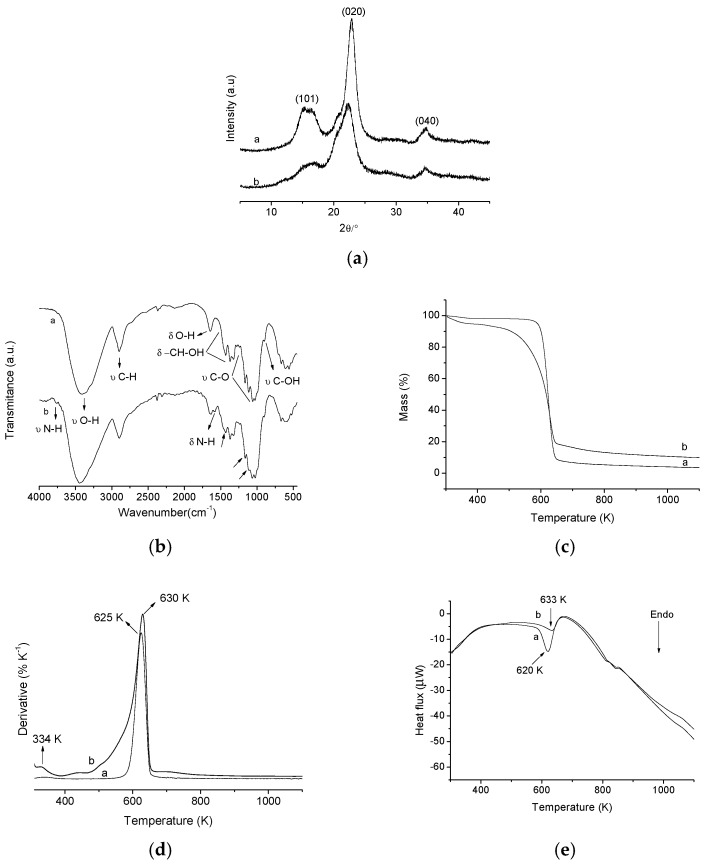
Graphs of X-ray diffraction (XRD) (**a**); fourier transform infrared spectroscopy FTIR (**b**); thermogravimetry (TG) (**c**); derived from thermogravimetry (DTG) (**d**) and differential scanning calorimeter (DSC) (**e**) of the pristine cellulose (**a**) and modified cellulose (**b**).

X-ray diffraction was used to observe the crystallinity of the cellulose before (a) and after (b) chemical modification by ethylenediamine as shown in Figure 2b. In the XRD diffractograms of pristine cellulose, three peaks can be observed at 15.54°, 22.90° and 34.72°, which are related to the crystallographic planes (101), (002) and (040), which are characteristic of pure microcrystalline cellulose, corresponding to interplanar distances of 5.64, 3.96 and 2.59 Å, respectively [22,23]. After the reaction, the CN (CN is the abbreviation for modified cellulose) biomaterial diffractogram showed the same crystallographic planes of pristine cellulose, however, there was a reduction in the intensity of these peaks, i.e., a decrease in crystallinity of the modified polymer, being more pronounced in crystallographic planes (101) and (002). This reduction in the cellulose crystallinity, after chemical modification, is attributable to the incorporation of amino groups on the cellulose surface. The crystalline regions of pristine microcrystalline cellulose are formed due to the intense hydrogen interactions of inter and intramolecular nature, which results in an ordered structure. Therefore, the incorporation of amino groups on the cellulose surface causes changes in hydrogen inter and intramolecular interactions of polymer, modifying its own organizational structure and thus decreasing the crystallinity of the cellulose after chemical modification [8,24].

Furthermore, to quantify the cellulose crystallinity reduction, after chemical modification, the cellulose crystallinity index (*IC*) was determined by the method proposed by Segal et al. [25]. The IC was calculated as shown in Equation (1) below:

*IC* = [(*I*_002_ − *I_am_*)/*I*_002_] × 100 (1)

where *I*_002_ is the maximum intensity of refraction plane (002) (22° ˂ 2θ ˂ 23°) and *I_am_* is the amorphous diffraction intensity (18° ˂ 2θ ˂ 19°). Thus, an *IC* = 83.71% for pristine cellulose was obtained while the modified cellulose showed an *IC* = 69.10%. This reduction in cellulose *IC*, after chemical modification, agrees with the results mentioned above, showing that the incorporation of amino groups on the surface of the cellulose causes a disturbance in the hydrogen inter and intramolecular bonds, thus reducing the crystallinity of the polymer.

The elemental analysis results of pristine microcrystalline cellulose and CN are shown in Table 1. The modification of cellulose with ethylenediamine generated a modified polymer with 1.10 ± 0.01% nitrogen incorporated into its surface. Based on this value, the amount of nitrogen covalently bonded to the cellulose surface is 0.78 ± 0.02 mmol per gram of polymer, which equals 0.39 ± 0.01 mmol of ethylenediamine per gram of polymer. These results confirm that the reaction between cellulose and ethylenediamine was efficient.

Although there are in literature works related to the reaction between cellulose and ethylenediamine, which in the amount of nitrogen incorporated into the cellulose surface is greater than the value presented in this work [10,24,26], our synthesis route is more appropriate for two main reasons: it is environmentally and economically more viable than those of previously cited works, as in these works, an intermediate reaction (halogenation) through chlorination of cellulose is used, in order to increase the reactivity of the cellulose hydroxyls, however, the reagent used in this step (thionyl chloride) is toxic, quite expensive and the generated product (chlorinated cellulose) can also affect the environment, making this a less desirable approach.

Infrared spectroscopy is an important information source for the qualitative evaluation of groups incorporated on cellulose and can confirm the changes on the surface of modified polymers [8]. The pristine cellulose spectrum is shown in Figure 2a, line (a) where the presence of O–H groups can be observed by the band at 3413 cm^−1^, which is related to the stretching vibrations of the O–H bonds from the aromatic ring. Another important vibration present in the spectrum of pristine cellulose occurs near 2898 cm^−1^, this vibration is related to stretching vibrations of C–H groups. The band at 1642 cm^−1^ corresponds to bending vibrations of the primary and secondary O–H groups present in the cellulose structure. The region between 1500 cm^−1^ and 1200 cm^−1^, shows the presence of bands that also correspond to the deformation of primary and secondary O–H groups. In the region between 1200 and 1000 cm^−1^ the stretching vibrations of alcoholic groups (C–O) occur. Finally, the bands presented under 1000 cm^−1^ are attributed to the absorptions of alcohol groups [8,22,23].

The FTIR spectrum of CN polymer (Figure 2a, line (b)) presents several bands similar to those present in FTIR spectrum of pristine microcrystalline cellulose, but the hydroxyls, present in the cellulose, exhibit regions of intense absorptions similar to that of the incorporated amino groups, then, these bands overlap, thus hindering the viewing of the absorption bands related to the amino groups.

Nevertheless, significant changes after cellulose modification by ethylenediamine are observed as the appearance of two bands, one around 3764 cm^−1^, related to the N–H stretching of primary amines and another one at 1575 cm^−1^, corresponding to N–H deformation primary amines. It is observed that decreases in band intensities at 1430 cm^−1^, 1157 cm^−1^ and 1108 cm^−1^ occur, which is due to a reduction of the number of O–H vibrations due in turn to the replacement of O–H groups by amino groups after the chemical modification step [26,27].

Thermogravimetric curves of the pristine cellulose (a) and modified cellulose (b) are shown in Figure 2c and the derivatives of the curves (DTG) are given in Figure 2d. For pristine cellulose, the curve shows a single decomposition event between 574 K and 649 K, which corresponds to a mass loss of 88.90% and a maximum decomposition temperature t*_peak_* at 625 K. This event is associated with cellulose decomposition. However, there is a mass loss of 1.47% in the temperature range between 315 K and 377 K, corresponding to the water physically adsorbed on the cellulose surface. Moreover, it is observed that the cellulose is not completely decomposed at the temperatures described above, considering that until 1100 K, there are approximately 3.37% of remaining residue [22,23,26]. The TG curve, from the modified polymer, presented two events of decomposition. The first is observed between 304 K and 364 K with a mass loss of 4.45%, showing t*_peak_* at 334 K, which is related to physisorbed water on the surface of the polymer. The second decomposition event occurs between 456 K and 651 K, corresponding to a mass loss of 74.31%, and t*_peak_* at 630 K, which is related to decomposition of the modified polymer, i.e., decomposition of the amino groups and the cellulose structure [10,26].

These results indicate that CN polymer is more stable than the pristine microcrystalline cellulose at temperatures above 630 K. In other words, the immobilization of amino groups on the surface of the cellulose makes the polymer more thermally stable and, therefore, a lower mass loss is observed at temperatures above 630 K [26].

The DSC curves (Figure 2e) confirm the change of the thermal stability of the cellulose after chemical modification. The DSC of pristine cellulose has an endothermic peak at 620 K associated with the same event seen in the TG analysis (cellulose decomposition). In the DSC curves of modified cellulose an endothermic peak associated with decomposition of cellulose incorporated with amino groups is also observed, however, this peak is at a greater temperature (633 K) than that of pristine cellulose, thus confirming the increased thermal stability of the modified cellulose compared to pure cellulose at temperatures above 630 K.

**Figure 3:**
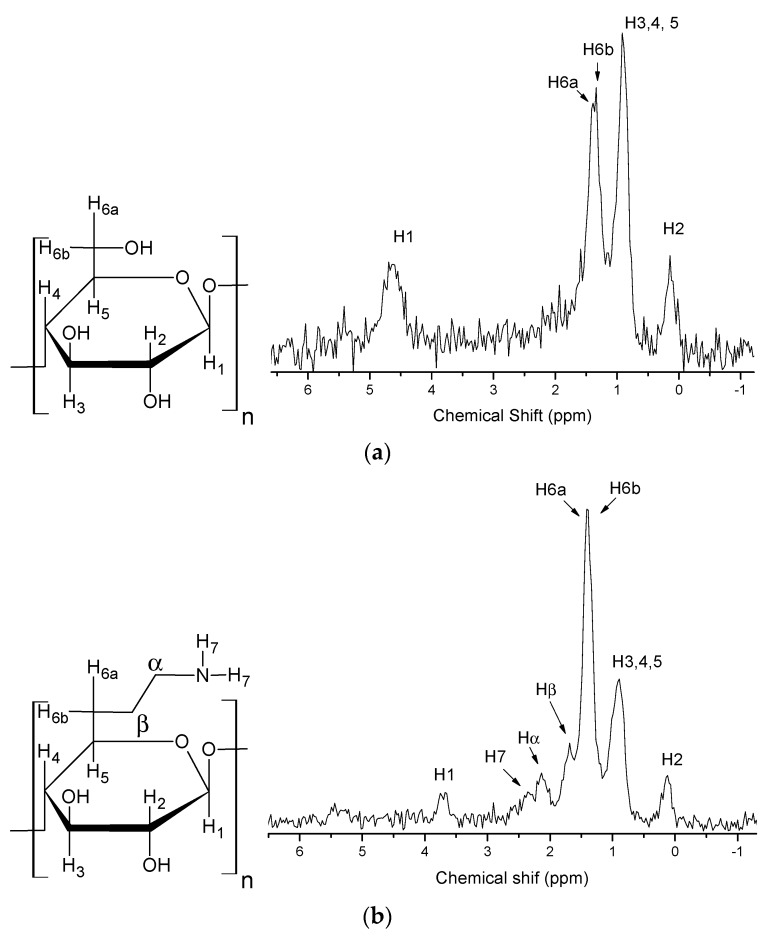
Solid state ^1^H-NMR spectra of pristine microcrystalline cellulose (**a**) and CN polymer (**b**).

Figure 3a shows the structure of pristine cellulose and the solid state ^1^H-NMR spectrum. In this figure the chemical shifts of each proton present in the structure of pure microcrystalline cellulose can be observed. The protons (H6a and H6b) of the C6 methylene group appear at 1.40 ppm and 1.33 ppm. This splitting is due to the spin-spin coupling between the two vicinal protons at C6. The other chemical shifts are: H1 (4.67 ppm), H2 (0.13 ppm) and H3,4,5 (0.90 ppm) [28].

The solid state ^1^H-NMR spectrum of the CN cellulose in Figure 3b shows changes in cellulose structure after incorporation of ethylenediamine. The H1 signal moves from 4.67 ppm to 3.70 ppm, due to the depolymerization process of the amorphous portion [8], and a decrease of the intensity of the peak at 0.90 ppm relative to H3, H4 and H5 is also observed. In the spectrum signals at 2.39 ppm, 2.15 ppm and 1.71 ppm appear, related to the protons of the amine group (H7) and the protons from methylene groups (α and β), respectively [27].

**Figure 4:**
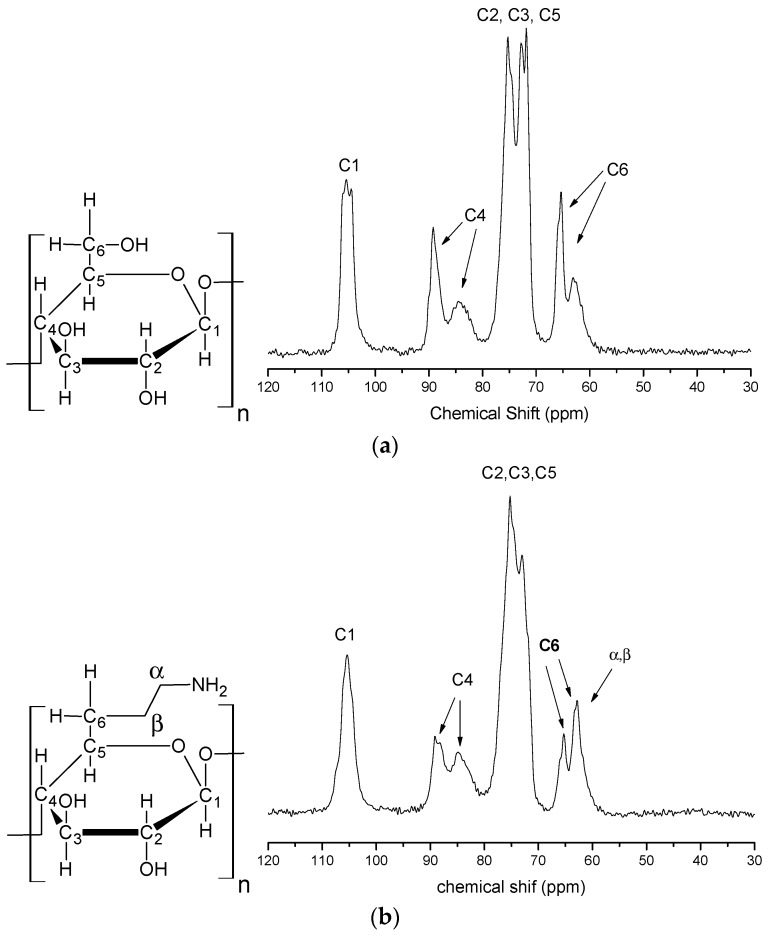
Solid state ^13^C-NMR spectra of pristine microcrystalline cellulose (**a**) and CN polymer (**b**).

Figure 4a shows the solid state ^13^C-NMR spectrum of the pristine cellulose. In this spectrum the carbon that exhibits the highest chemical shift is the C1 (carbon 1) one at 105 ppm because it is bonded to two oxygen atoms. The signals observed at 89 and 84 ppm are assigned to C4, which is connected to only one oxygen, which is responsible for the 1,4-β-glucoside linkage. The signal at 89 ppm indicates a region of higher crystallinity and the signal at 83 ppm represents a less-crystalline or amorphous carbon. The signals observed at 75 ppm, 73 ppm and 71 ppm are attributed to C2, C3 and C5, which have equivalent chemical environments, this is, they are all secondary carbons linked to CH groups. Lastly, the signals observed at 65 ppm and 63 ppm are assigned to C6, this carbon presents the lowest chemical shift because is a primary carbon connected to a hydroxyl and is the single CH_2_ present in the cellulose. The signal that appears at 65 ppm is related to regions of greater crystallinity and the signal in 63 ppm is related to regions of lower crystallinity [8,27].

The spectrum of modified cellulose is presented in Figure 4b. No significant change in the C1 signal relative to that of pristine cellulose is evident. However, this spectrum shows significant changes in the other chemical shifts relative to those of pristine cellulose. In the region from 75 ppm to 71 ppm, related to C2, C3 and C5, there was the disappearance of the peak in 71 ppm and a decrease in intensity of the 72 ppm peak. At C4 after chemical modification there was a decrease in intensity of the 89 ppm peak, which indicates a region of higher crystallinity. This occurred due to the depolymerization process of the crystalline portion. For the C6 peak there was a decrease in intensity of the 65 ppm peak too, which occurred because of the depolymerization of the crystalline part of the cellulose; these changes therefore prove the immobilization of ethylenediamine and the formation of CN polymer. In addition, the presence of the α and β carbons of ethylenediamine cannot be identified in this spectrum due to the fact the region of these chemical shifts, from 40 ppm to 80 ppm, is a region where cellulose already has very intense signals [8,10,27]. These results also confirm the XRD results showing that there is a decrease in cellulose crystallinity after the chemical modification with ethylenediamine.

### 2.2. Reaction Mechanism and Theoretical Calculations

**Scheme 1:**
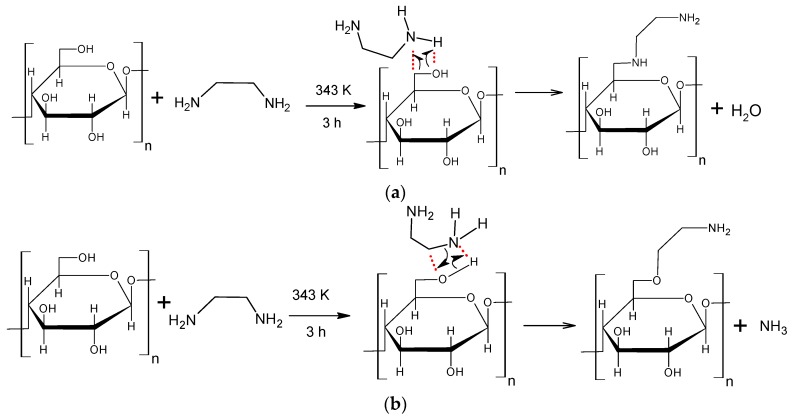
Reaction mechanism proposed for the reaction between cellulose and ethylenediamine and the possible formed products.

The reaction between cellulose and ethylenediamine can occur mainly by two routes, creating different products. This reaction occurs in a concerted manner, that is, it takes place in one step, with the rupture and the formation of chemical bonds occurring simultaneously. The reaction temperature and alkalinity of the reactants are the two main factors influencing this reaction. The temperature provides energy to the system to convert reactants into products, i.e., the temperature helps to overcome the reagents/products energy barrier. The alkalinity of the reagent, cellulose (alcohol) and ethylenediamine (amine), is very important to determine what products are formed. The proposed mechanism a (Scheme 1a), is based on the interaction between the hydroxyl of the cellulose C6 (since this is the most reactive hydroxyl of cellulose) and the hydrogen of the amino groups of ethylenediamine, releasing water and forming the modified cellulose with two amino groups incorporated into its structure. However, as the amine (ethylenediamine) is more alkaline than the alcohol (cellulose) [29], the more likely is that the nitrogen from the amino groups of ethylenediamine interacts with the hydrogen of the hydroxyl in cellulose C6, releasing ammonia and forming cellulose with an amino group incorporated on its surface, as shown in Scheme 1b.

**Figure 5:**
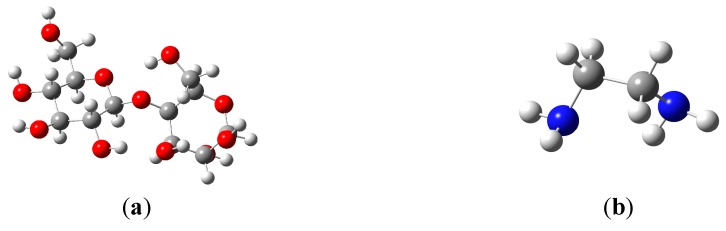

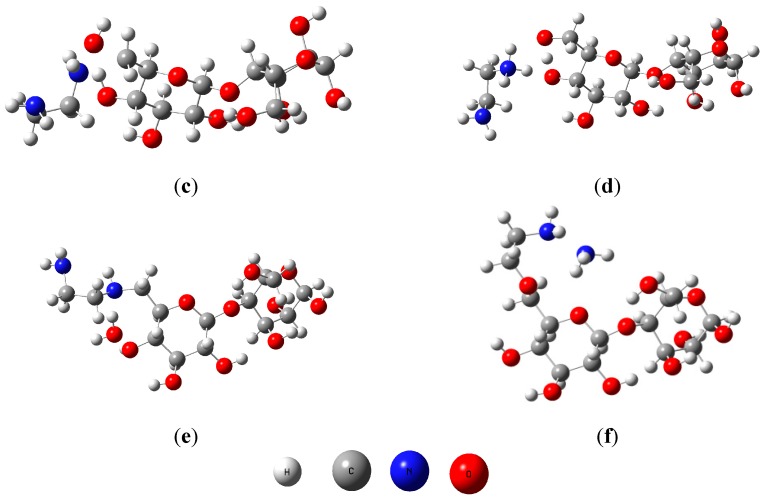
Geometries of cellulose dimer (**a**); ethylenediamine (**b**); transition states (TS_A_, (**c**) and TS_B_, (**d**)) and products (P_A_ (**e**) and P_B_ (**f**)).

To determine which the proposed reaction mechanism (a and b) is the most stable and favorable for this reaction, were performed computations at level B3LYP/6-31G(d). Reactants, transitions states and adducts were characterized by frequency calculations, followed by intrinsic reaction coordinate method. Figure 5 shows the geometries obtained for reactants, transition states and products.

**Figure 6:**
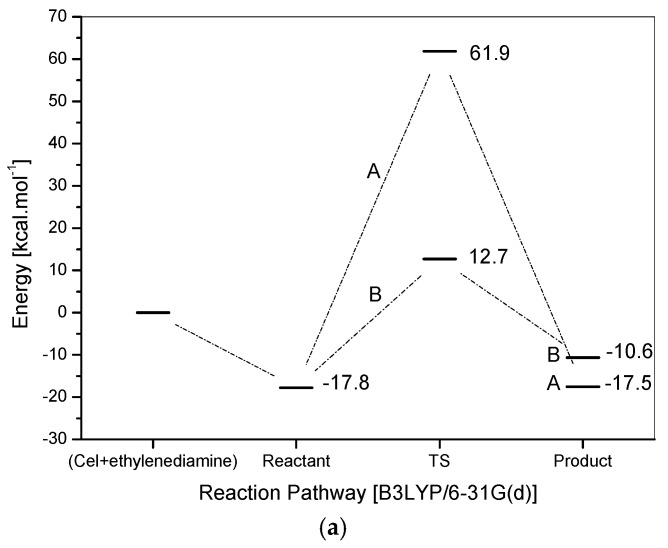

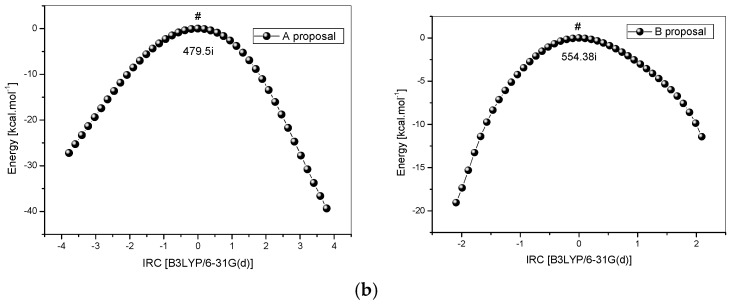
(**a**) Energy profile for the reaction between cellulose and ethylenediamine; (**b**) IRC for the reaction between cellulose and ethylenediamine, route A (left) and route B (right).

Energy profiles for the two proposed mechanisms are shown in Figure 6a. The computations support both proposed mechanisms. Initially, it can be seen that the products, generated by two mechanisms, are more stable than the reactants before the reaction starts, where P_A_ is −17.5 kcal·mol^−1^ more stable than the reactants and P_B_ is −10.5 kcal·mol^−1^ more stable than the reactants, which confirms that this reaction tends to happen. Another important point is the reaction energy requirement, i.e., the reaction needs heat to overcome the reagents/products barrier energy and then occurs. Due to this, the experimental reaction exposed to the influence of temperature. The product (P_A_) is approximately 7 kcal mol^−1^ more stable than the product (P_B_).

However, in this case, the kinetics define the more favorable route, due to a lower barrier, pointing to mechanism B as the most probable one to occur. The difference in energy between the barriers is almost 50 kcal mol^−1^. If the sub-products (water-proposal A and ammonia-proposal B) formed are not considered, in the ethylenediamine and cellulose dimer reaction, product B is about 12,500 kcal·mol^−1^ more stable than the analogous one formed in the proposal A. This result is quite interesting because it can justify the difficulty of interaction that leads to a high level of energy for the TS_A_, showing that in fact the proposal B, doubtlessly, is the most plausible one among the two alternative proposals.

Although ethylenediamine is a bidentate ligand, the distance of around 6 Å between the two reaction sites (Cel-CH_2_OH), in the studied dimer, suggests that only a single coordination occurs as the distance of groups (–NH) in ethylenediamine is no more than 3 Å. The frequency calculations showed only one imaginary frequency to the maximum found via QST3, and IRC calculations (Figure 6b) have also shown a good correspondence with the reaction routes.

### 2.3. Adsorption Test

#### 2.3.1. Point of Zero Charge (pH_pzc_)

**Figure 7:**
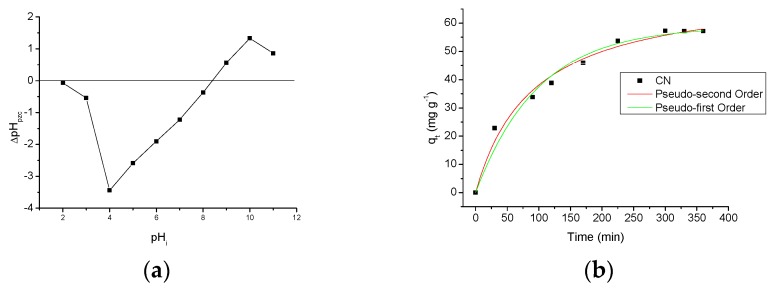

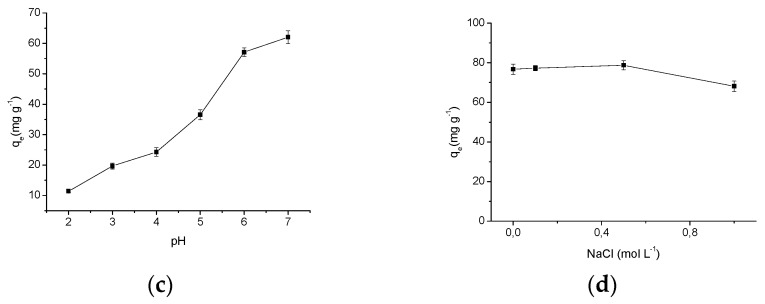
(**a**) Figure of the point of zero charge from the CN polymer; (**b**) contact time effect in the AMI adsorption by CN polymer and the theoretical kinetic models; (**c**) effect of pH in the AMI adsorption by CN polymer; (**d**) effect of NaCl concentration in the AMI adsorption by CN polymer.

Figure 7a shows a graph of the zero charge potential, pH_pzc_ of CN polymer. This graph shows how the pH of the medium affects the surface of the adsorbent [8,9]. By analysis of the graph, it can be observed that the pH_pzc_ of the CN polymer is 8.40 and at low pH values, the surface of the polymer retains a small amount of the aqueous medium protons, whereas when the pH increases, this retention amount increases until the pH_i_ = 4, i.e., the pH_f_ from aqueous medium will be greater than the pH_i_. From the pH_i_ 5, this retention decreases with the medium pH to approximately pH 8.40, where positive and negative charges are equivalent. Above pH 8.40, the surface of the polymer starts releasing protons to the solution, and since this release increases with the increase of pH until pH_i_ = 10, this indicates the presence of the polymer in the aqueous medium makes the pH_f_ smaller than the pH_i_. After this point, pH = 11, the proton retention decreases. This result shows that the pH of the medium affects the surface of the CN polymer, meaning that ions in the solution (H^+^ or OH^−^) can interact with the active sites of the CN polymer, thereby changing the charge balance of this polymer [3]. This result also shows that the incorporation of amino groups, on the cellulose surface, changed the pH_pzc_ graph of CN polymer compared to the pristine microcrystalline cellulose at different pH values. This change in the pH_pzc_ graph of pristine cellulose can be observed when comparing with some studies in literature which show similar results [22] to the pH_pzc_ graph of the modified cellulose present in this study. This confirms the presence of amino groups on the surface of the cellulose after chemical modification, and also indicates that these groups change the properties related to adsorption of the adsorbent.

#### 2.3.2. Time Effect

The adsorption kinetics is one of the most important characteristics that influence the solute adsorption rate, and determines the adsorption efficiency of the adsorbent [30]. Figure 7b describes the AMI adsorption behavior in the CN polymer as a function of time and the theoretical kinetic models. For this graph, it can be observed that after 300 min of contact between the drug and the polymer, the amount of AMI adsorbed became substantially constant, with maximum adsorption is 57.31 ± 1.00 mg·g^−1^. This indicates that, after 300 min there are no more active sites available in polymer to interact with the drug molecules, because all active sites are already occupied by drug molecules, avoiding the AMI adsorption even the adsorptive process contact time be increased, and the system reaches saturation equilibrium.

The adsorption kinetics experimental data were adjusted to the pseudo-first order and pseudo-second order models. The kinetic parameters obtained for both models can be seen in Table 2, where it is observed that the kinetic model that presented the best fit to the adsorption process was the pseudo-second order one, because showed a higher correlation coefficient (*R*^2^ = 0.9854). The best correlation for the system provided by the pseudo-second order model suggests the reaction that occurs in the adsorbent surface is the step that controls the adsorption speed, and this chemisorption occurs involving valence forces by sharing or exchange of electrons between the adsorbent and the adsorbate [31,32,33].

#### 2.3.3. Effect of pH

**Figure 8:**
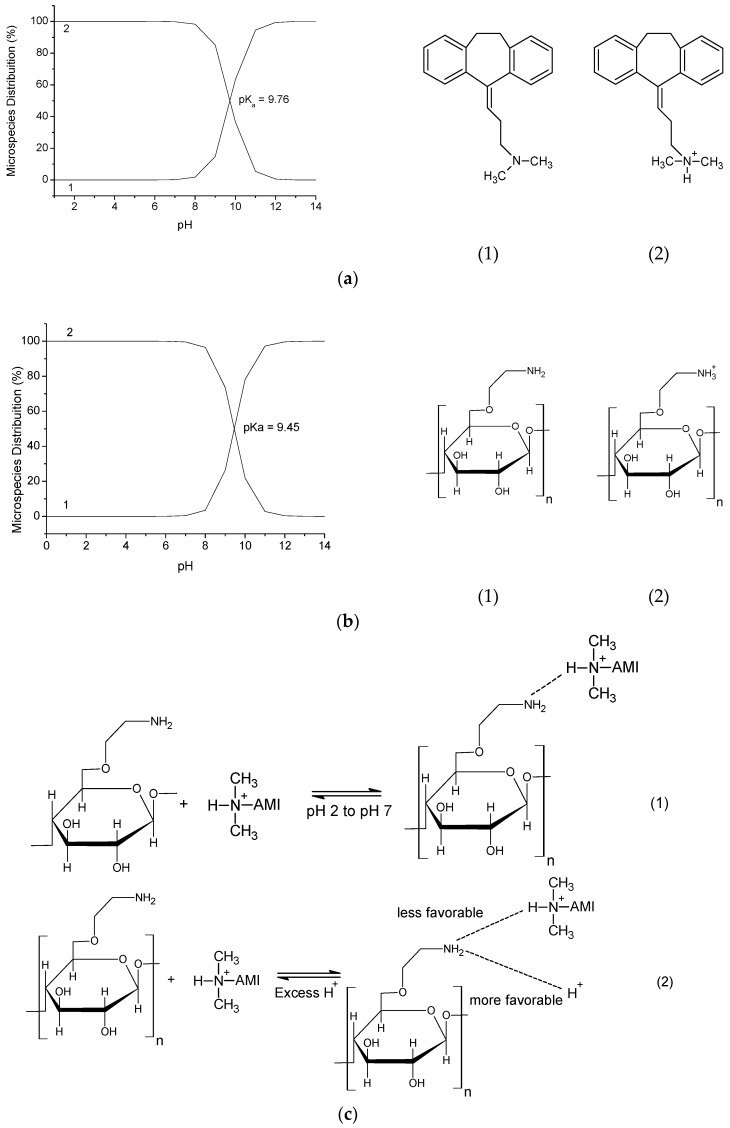
(**a**) Distribution of AMI microspecies at different pH values; (**b**) distribution of CN polymer microspecies at different pH values; (**c**) adsorption mechanism proposal of AMI adsorption by CN polymer.

The CN polymer was applied to AMI adsorption experiments varying the pH to determine the pH of maximum adsorption and the adsorption process behavior with respect to change in the pH. In Figure 7c it is observed that as the pH increases the amount of AMI adsorbed by the CN polymer increases, with a maximum adsorption, q_e_ = 62.06 ± 2.10 mg·g^−1^ at pH 7. This behavior can be explained by the interactions that can occur between the drug and the CN polymer. The drug has a p*K*_a_ = 9.76 (the p*K*_a_ values and the distributions of AMI and the polymer microspecies were obtained using the software MarvinSketch 4.15.13 (ChemAxon, Budapest, Hungary)), which shows that at pH under the p*K*_a_ there is a predominance of AMI microspecies with the protonated amino group, as shown in Figure 8a. Thus, in all pH values investigated (pH 2 to pH 7) the drug is protonated, i.e., positively charged.

The CN polymer which has also an amino group in its structure, which has a p*K*_a_ = 9.45, where this polymer presents the prevalence of protonated microspecies at pH values below its p*K*_a_ value, as shown in Figure 8b. Thus, it can be seen that the AMI adsorption at CN polymer surface occurs due to the hydrogen interactions that can be formed between the amino groups from both components, as shown in Figure 8c(1). Therefore, the increased adsorption caused by the increase of pH may be explained by the excess of H^+^ ions at lower pH values, which may interact with polymer active sites (pH_pzc_ proved that both H^+^ and OH^−^ ions, from aqueous medium can interact with CN polymer surface), since they are smaller than the drug molecules, thus, protonating the CN polymer (Figure 8c(2), so at low pH values, both the drug and the polymer are positively charged, which will promote an electrostatic repulsion between them, making the adsorption more difficult. As the pH increases, the amount of H^+^ ions decreases, and thus decreases the polymer protonation, facilitating the drug adsorption on the polymer surface [23,34,35].

The CN polymer presented more efficient AMI adsorption in aqueous media than unmodified polymer. A previous study showed that the maximum adsorption capacity of pristine cellulose was 20.23 ± 0.80 mg·g^−1^ at pH 5 and 298 K [3]. Thus, incorporation of amino groups on cellulose has promoted an increase in the maximum adsorption capacity of 206.77% (CN polymer: q_e_ = 62.06 ± 2.10 mg·g^−1^ at pH 7 and 298 K). Furthermore, at all pH values investigated, the modified polymer presented an adsorption capacity higher than pristine microcrystalline cellulose, which shows and confirms that the CN polymer is more effective as sorbent than pristine microcrystalline cellulose.

#### 2.3.4. Effect of Concentration and Temperature

**Figure 9:**
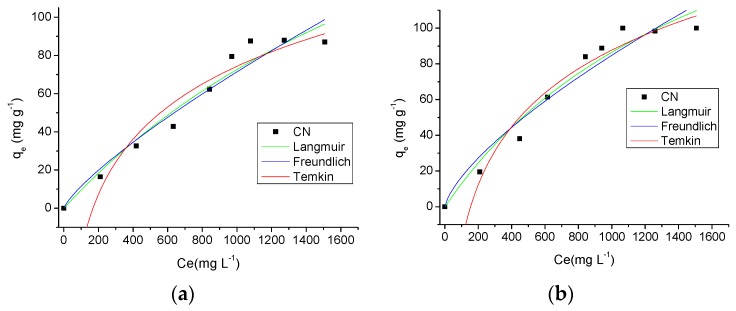

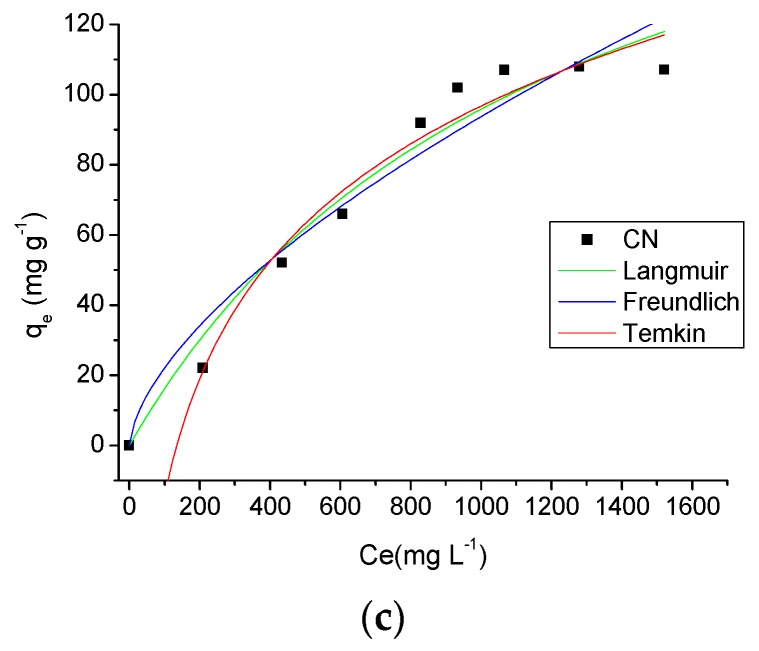
Effect of concentration and temperature (298 K (**a**), 308 K (**b**) and 318 K (**c**)) in the AMI adsorption by CN polymer and theoretical models of isotherms.

The adsorption isotherms provide fundamental physico-chemical data for evaluate the adsorptive capacity of an adsorbent [36]. The isotherms obtained for the three temperatures studied (298 K, 308 K and 318 K) and theoretical models of isotherms can be seen in Figure 9. From the analysis of these graphs, it is observed the temperature influenced the adsorption process, and as the temperature increased the adsorption capacity of AMI by the CN polymer was also increased. Another important factor influencing the adsorption was the drug solution concentration. As the drug solution concentration was increased an increase in the amount of drug adsorbed by the adsorbent occurred, with a maximum adsorption of 87.66 ± 0.60 mg·g^−1^, 99.99 ± 2.50 mg·g^−1^ and 107.07 ± 1.43 mg·g^−1^ at 298 K, 308 K and 318 K, respectively.

In this study, the isotherm experimental data were processed according to the Langmuir, Freundlich and Temkin models, and the results are shown in Table 3. Using the correlation coefficient values, *R*^2^, the isotherm model at a temperature of 298 K which showed the best fit to the adsorption data was the Langmuir model (*R*^2^ = 0.9538 at 298 K), while for the temperatures of 308 K and 310 K, the model with the best fit to the adsorption process was the Temkin model (*R*^2^ = 0.9652 at 308 K and *R*^2^ = 0.9734 at 318 K). The Langmuir model assumes that adsorption takes place at specific homogeneous sites within the adsorbent by monolayer adsorption without any interaction between adsorbed molecules [37]. However, the Temkin model contains a factor that considers the adsorbent-adsorbate interactions, suggesting that because of these interactions the heat of adsorption of all molecules linearly decreases as the surface of the adsorbent is covered [37,38].

Thermodynamic parameters [39] (Δ*H*°, Δ*S*° and Δ*G*°) obtained from the experimental data are shown in Table 4. The positive value of Δ*H*° indicates an endothermic adsorption process. The magnitude of Δ*H*° value shows that the adsorption process is a physisorption (2.1 to 20.9 kJ mol^−1^) or chemisorption (80–200 kJ mol^−1^), therefore, by the Δ*H*° value (7.7095 kJ mol^−1^) can be observed that AMI adsorption by the CN polymer is a physisorption [40]. The positive value of Δ*S*° shows that there was an increase in randomness at the solid/liquid interface during the adsorption process of AMI [36,40]. Positive Δ*G*° values reflect the non-spontaneous nature of AMI adsorption process at the studied temperatures. Moreover, it is observed that as the temperature rises, there is a decrease in the Δ*G*° value, i.e., the spontaneity of the adsorption process is increased. This indicates that the adsorption becomes more favorable at higher temperatures, and consequently, the increase in temperature causes an increase in the amount of drug adsorbed by the CN polymer [36,41]. These results indicate that temperature is an important parameter in the adsorption process of AMI from aqueous medium using as adsorbent the CN polymer.

#### 2.3.5. Ionic Strength

To verify if the presence of ions in solution influence the drug adsorption process by the CN polymer, adsorption tests were performed by varying the NaCl concentration (ionic strength) in the solution as shown in Figure 7d. Through the graph, it can be seen that the increase of NaCl concentration does not significantly change the adsorption of the drug by CN polymer, being found that only at 1.0 mol·L^−1^ of NaCl there was a small decrease in the amount of drug adsorbed by the CN polymer. This result shows that the Na^+^ ions have a minimal influence in the drug adsorption by CN polymer, and a slight decrease in the adsorption occurs in a solution of a higher NaCl concentration due to the increased hydrophobic interactions of AMI molecules caused by an increase in the ionic strength of the solution. These interactions overcome the repulsive electrostatic interactions, and favor the aggregation of the drug, thus preventing the interaction between AMI and the polymer. These results confirm that the predominant interactions in the AMI adsorption by CN polymer are hydrogen interactions [2,42].

### 2.4. Drug/Polymer Interaction

**Figure 10:**
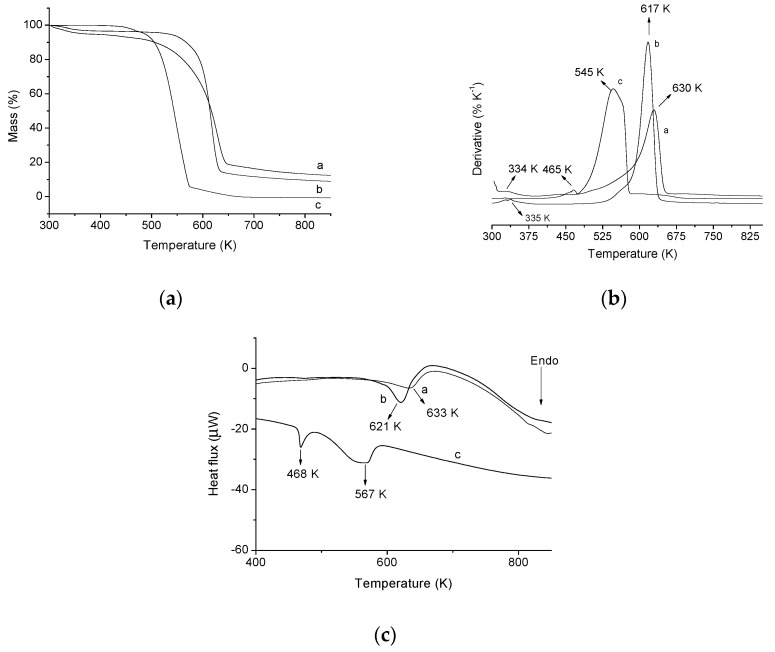
(**a**) Thermogravimetric curve (TG) of CN polymer before (**a**) and after (**b**) AMI adsorption and pure AMI (**c**); (**b**) derived from thermogravimetric curve (DTG) of CN polymer before (**a**) and after (**b**) AMI adsorption and pure AMI (**c**); (**c**) DSC of CN polymer before (**a**), after (**b**) and AMI adsorption and pure AMI (C).

To confirm if AMI is really adsorbed on the CN polymer surface, after the adsorption tests, in the best conditions (t = 300 min, pH 7, drug solution concentration = 1000 mg·L^−1^ and T = 298 K), the solid was dried and characterized by TG/DTG/DSC, as shown in Figure 10. From the TG/DTG graph of pure drug, three stages can be seen in the decomposition process. The first step occurs at a maximum temperature of 465 K. This decomposition stage is related to the onset of drug decomposition and its fusion process. The AMI fusion process can be confirmed by the DSC graph, which it is observed an endothermic peak at 468 K, related to this fusion process [43]. The second step occurs with a mass loss of 90.20% in the range of 480 K to 574 K and the maximum decomposition temperature at 545 K. This step is related to AMI decomposition. This event can be observed in the DSC curve of the drug, which presents an endothermic peak at 564 K, also related to the AMI decomposition. Finally, the third step takes place at temperatures higher than 575 K, and is related to the step of complete decomposition of AMI [44,45].

In the TG/DTG graph of CN polymer (Figure 10) significant changes in the thermal stability of the polymer can be seen, due to the presence of AMI on the cellulose surface. After adsorption, the same two events of the absorption process of the CN polymer before the adsorption can be observed. However, as the drug is adsorbed on the surface of the polymer it is possible to observe changes in percentage terms of loss due to the initial decomposition temperature and mass from each step. These changes show that drug decomposition occurs between the temperatures range of 520 K to 635 K, because in this temperature range significant changes are observed in the TG curves. In addition, the main drug decomposition event happens within this range, confirmed by the drug decomposition maximum temperature (454 K) in the DTG of the drug. Finally, AMI presence on the surface of the CN polymer is also confirmed by DSC, which shows that the displacement of the endothermic peak from 633 K to 621 K. The temperature of 621 K is related to the decomposition both drug as the CN polymer. Therefore, these results taken together confirm that the CN polymer actually adsorbed AMI drug on its surface.

## 3. Materials and Methods

### 3.1. Materials

Pure microcrystalline cellulose (Fagron, São Paulo, SP, Brazil), ethylenediamine (Aldrich, São Paulo, SP, Brazil), sodium hydroxide (Synth, São Paulo, SP, Brazil), hydrochloric acid (Synth), potassium nitrate (Química Moderna Ind, Barueri, SP, Brazil), amitriptyline HCl (Pharma Nostra Comercial Inc., Anápolis, GO, Brazil) and deionized water were used. All reagents were analytical grade and no previous purification was need.

### 3.2. Cellulose Chemical Modification with Ethylenediamine

Cellulose (1.0 g) was modified with ethylenediamine (10.0 mL, 1:25 cellulose/ethylenediamine molar ratio) in the absence of solvent for 3 h, under stirring and at 343 K. Subsequently, the product was washed with distilled water, centrifuged (3500 rpm for 5 min, Centrifuge model tand, Nova Instruments Piracicaba, SP, Brazil) and the supernatant was removed and the polymer was oven dried for 12 h at 333 K. The modified polymer is a water insoluble powder and was named CN. The yield in the production of CN polymer was 71.67%. This methodology has been obtained from other studies [10,24], but with significant changes, which are related to the steps of the chemical modification of the cellulose surface. Most studies deal with the modification of the cellulose surface using an intermediate halogenation reaction, through chlorination of cellulose, in order to increase the reactivity of the cellulose hydroxyls. Considering that the reagent used in this step (thionyl chloride) is toxic, in this study the modification of cellulose with ethylenediamine is carried out without the halogenation step, making the reaction less harmful to the environment.

### 3.3. Characterizations

X-ray diffraction was performed on a D600-XR instrument (Shimadzu, Kyoto, Japan), at 2θ in the range of 5–75°. The scan speed was 8.33 × 10^−2^ s^−1^ using the Cu Kα radiation (λ = 154 pm). Elemental analysis was performed in a Perkin Elmer 2400 series II elemental analyzer (Perkin Elmer, Waltham, MA, USA). The absorption spectra in the infrared (FTIR) were obtained using a 660 IR spectrophotometer (Varian, Palo Alto, CA, USA) by the 1% KBr pellet method, with an accumulation of 32 scans over the range of 4000–400 cm^−1^. Thermal analysis (TG/DTG/DSC) was performed using a Q600 V20.9 Build TA instrument (TA instrument, New Castle, DE, USA) under a nitrogen atmosphere at a flow rate of 100 mL min^−1^ and a heating rate of 10 °C min^−1^. Solid state NMR experiments were conducted using an Avance spectrometer (Bruker, Billerica, MA, USA,) at a magnetic field of 9.4 T. The ^13^C{^1^H}CP-MAS (Cross-polarization magic angle spinning) spectra were measured under spinning at 10 kHz, using a CP contact time of 2.0 ms and relaxation delay of 2 s. All spectra were acquired with TPPM (two-pulse phase-modulated) proton decoupling during the data acquisition applying decoupling pulses. The ^1^H-NMR experiments were acquired under spinning at 14 kHz, using a spin echo sequence, with synchronized spinning and a saturation train of 56. Chemical shifts are reported relative to TMS referencing standard. The AMI concentration was obtained in an ultraviolet-visible spectrophotometer (Cary 300 Varian, Palo Alto, CA, USA, λ = 239 nm).

### 3.4. Adsorption

#### 3.4.1. Point of Zero Charge (pH_pzc_)

The point of zero charge (pH_pzc_) was determined by the method of adding solids [22]. Thus a KNO_3_ solution (50.0 mL, 0.1 mol·L^−1^) were added to a series of flasks. The initial pH (pH_i_) of each flask was adjusted from 2 to 11 by adding solutions of 1.0 mol·L^−1^ HCl and/or NaOH. Then, one aliquot of 20.0 mL of each flask (pH = 2 to pH = 11) was transferred to a 125 mL Erlenmeyer containing CN polymer (20.0 mg), which was placed under stirring for 24 h, at 298 K. Finally, the supernatant was centrifuged (3500 rpm for 5 min) and the final pH measurements (pH_f_) were performed. The difference between the pH_i_ and pH_f_ is the ΔpH_pzc_, and was calculated by Equation (1) [8], being that the pH_pzc_ is the pH at which the amount of negative charges and positive charges is equal:

ΔpH_pzc_ = pH_i_ − pH_f_ (2)

#### 3.4.2. Influence of Time

The kinetic study of AMI removal was carried out in batch process. Initially, 20.0 mL of a drug solution (1000 mg·L^−1^) without pH adjustment (6.6 ± 0.1) was placed in contact with approximately 20.0 mg of CN. After, the solutions were placed under stirring, at 298 K, and varying the contact time. Subsequently, the supernatant was separated by centrifugation at 3500 rpm for 15 min [9]. The drug concentration was determined for each time by UV/Vis from calibration curves at a wavelength λ = 239 nm, which corresponds to the length of maximum absorption of the drug (all the experiments were in triplicate, with limit of detection 1.0 mg L^−1^), and the amount adsorbed of the adsorbent, *q* (mg·g^−1^) was calculated by Equation (3) [3]:

(3) $$q=\frac{V(C_{0}-C_{f})}{m}$$

where *V* (L) is the volume of the drug solution, *C*_0_ (mg·L^−1^) is the initial concentration of the drug solution, *C_f_* (mg·L^−1^) is the concentration of the drug solution after adsorption at each time, *t* and *m* (g) the mass of the adsorbent.

From the time isothermal, the experimental data were adjusted to three kinetic models: pseudo-first order and pseudo-second order. The pseudo-first-order model is defined by Equation (4) [46]:

(4) $$q_{t}=q_{e,cal}\left[1-\exp(-k_{1}t)\right]$$

where *q_e_*,*_cal_* (mg·g^−1^) is the drug adsorbed amount at equilibrium, *q_t_* (g·mg^−1^) is the drug adsorbed amount at time *t* (min) and *K*_1_ (min^−1^) is the rate constant of pseudo-first-order adsorption.

The pseudo-second-order model is expressed by the mathematical expressions Equation (5) [47]:

(5) $$q_{t}=\frac{k_{2}q_{e,cal}{}^{2}t}{1+q_{e,cal}k_{2}t}$$

where *K*_2_ is the pseudo-second-order constant rate (g·mg^−1^·min^−1^).

#### 3.4.3. Effect of pH

The influence of pH on the AMI adsorption by the CN polymer was studied using a drug solution (1000 mg·L^−1^), varying the pH by adding HCl and/or NaOH solutions (1.0 mol·L^−1^), in order to obtain solutions with the following pH values: 2–7 (from pH 8 was not possible to perform the adsorption tests, since the addition of NaOH made the solution cloudy, making it impossible to perform UV/Vis adequately). Then, a rate of 20.0 mL of the drug solution, after pH adjustment, was placed in contact with approximately 20.0 mg of the CN polymer in an Erlenmeyer flask. The suspensions were placed in agitation at 298 K, and in the saturation time obtained in the previous section. Finally, the supernatant was separated from the adsorbent by centrifugation (3500 rpm for 5 min) and the concentration determined by UV/Vis (λ = 239 nm). The amount of the drug adsorbed on the surface of the polymer, was obtained by Equation (3) [34].

#### 3.4.4. Study of Concentration and Temperature

Adsorption isotherms were determined by placing 20.0 mg of the polymer in contact with 20.0 mL of solution containing various concentrations of AMI, ranging between 100 and 1600 mg L^−1^. The adsorbent-drug system was kept under stirring at temperatures of 298 K, 308 K and 318 K, at the best pH for adsorption (determined in Section 2.3.3), as well as, the best saturation time. After stirring, the supernatant was separated by centrifugation at 3500 rpm for 5 min and the concentration determined by UV/Vis spectroscopy at λ = 239 nm. The amount of drug adsorbed is calculated by Equation (3) [9].

In this study, the experimental data were adjusted to Langmuir, Freundlich and Temkin models [3,34]. The equation representing the isotherm model proposed by Langmuir is represented by Equation (6) [48]:

(6) $$q_{e}=\frac{K_{L}q_{max}C_{e}}{1+K_{L}C_{e}}$$

where *q_e_* (mg·g^−1^) corresponds to the amount of drug adsorbed by the adsorbent, *C_e_* (mg·L^−1^) the equilibrium concentration of the drug solution, *K_L_* is a proportionality constant which includes the equilibrium constant and is related to the adsorption free energy, which corresponds to the affinity between the surface of the adsorbent and the solute, and *q_max_* (mg·g^−1^) is the maximum amount of drug that can be adsorbed.

The Langmuir parameters may be expressed in terms of a dimensionless separation factor, *R_L_*, defined by Equation (7), thus being able to evaluate the shape of the isotherm:

(7) $$R_{L}=\frac{1}{1+K_{L}C_{e}}$$

where *C_e_* (mg·L^−1^) is the highest equilibrium concentration and *K_L_* is the Langmuir constant. For a favorable adsorption, the *R_L_* values must be between 0 and 1 (0 < *R_L_* < 1), while, *R_L_* > 1 represents an unfavorable adsorption; *R_L_* = 1 represents a linear adsorption and *R_L_* = 0, the adsorption process is irreversible [49].

To adjust the experimental data to the Freundlich isotherm model Equation (8) is used [50]:

(8) $$q_{e}=K_{f}C_{e}{}^{\left(1/n_{f}\right)}$$

wherein *q_e_* and *C_e_* have the same meaning as in the Langmuir equation, *K_f_* is a constant related to adsorption capacity and *n_f_* is a constant related to the strength of adsorption and spontaneity of adsorption, *n_f_* values between 1 and 10 indicate favorable adsorption.

For the Temkin isotherm model we used Equation (9) [51]:

(9) $$q_{e}=\frac{RT}{n_{T}}\ln(K_{T}C_{e})$$

wherein *n_T_* indicates, quantitatively, the reactivity of energetic sites of material and *K_T_* is a constant that includes the equilibrium constant. This model considers the system close to the Langmuir model.

The thermodynamic parameters (Δ*G*°, Δ*H*° and Δ*S*°) were obtained for the adsorption processes at temperatures of 298 K, 308 K and 318 K, using Equations (10) and (11) [22,41,52,53]:

(10) $$\log K_{e}=\frac{\Delta S^{o}}{2.303R}-\frac{\Delta H^{o}}{2.303RT}$$

(11) $$\Delta G^{o}=\Delta H^{o}-T\Delta S^{o}$$

where *R* is the gas constant (8.314 J mol^−1^ K^−1^), *T* is temperature (K) and *K_e_* is the equilibrium constant at temperature *T*, calculated by Equation (12):

(12) $$K_{e}=\frac{q_{e}}{C_{e}}$$

where *q_e_* (mg·g^−1^) is the amount adsorbed at equilibrium and *C_e_* (mg·L^−1^) is the equilibrium concentration.

#### 3.4.5. Ionic Strength

In ionic strength tests NaCl concentrations of 0.1 mol·L^−1^, 0.5 mol·L^−1^ and 1.0 mol·L^−1^ were used to adjust the ionic strength. Initially, 5.0 mL of NaCl solution was added to 40.0 mL of drug solution (1000 mg·L^−1^), and then, the pH was adjusted to the best adsorption pH, found in this pH study (Section 2.3.3). Subsequently, 20.0 mL of the previous solution were placed in contact with 20.0 mg of the adsorbent under stirring at 298 K and in the best time of adsorption (Section 2.3.2). After stirring, the supernatant was separated from CN polymer by centrifugation (3500 rpm for 15 min), and the concentration was determined by UV/Vis spectroscopy at λ = 239 nm from previous calibration curve for AMI. The amount of drug adsorbed was calculated by Equation (2) [2,35].

### 3.5. Computational Methods

Computational calculations were performed to determine the reaction mechanism and confirmation of products generated during the reaction between cellulose and ethylenediamine. All calculations were executed using the Gaussian 09W software (Revision B.01, Wallingford, CT, USA) [54]. Equilibrium geometries were computed in gas phase by Density Functional Theory [55,56] using the hybrid functional B3LYP [57,58,59] and the basis set 6-31G (d) [60]. Transition states (TS) were obtained with the Synchronous Transit-Guided Quasi-Newton (*N* = 3) QST3 method, developed by Schlegel et al. [61,62]. The maximum obtained were characterized by frequency calculations and subsequent Intrinsic Reaction Coordinate calculations [63,64]. The graphics were plotted in Origin 6.0 (OriginLab Corporation, Northampton, MA, USA). It is known that cellulose is a linear polydisperse polysaccharide consisting of several units of d-glucose molecules linked via β-1,4′-glycosidic bonds. The studied model was limited only to the dimer to represent the cellulose.

## 4. Conclusions

The cellulose modification by ethylenediamine in the absence of solvent for direct immobilization was successfully performed. The synthesized product was analyzed by XRD, elemental analysis, FTIR, TG/DSC, solid state ^1^H-NMR and ^13^C-NMR and these characterization techniques confirmed the reaction. Computational DFT, QST3 and IRC calculations corroborated the effectiveness of the reaction, besides showing which products formed in this reaction are the most stable. The CN polymer was efficient in the adsorption of AMI in aqueous medium. The adsorption process of the AMI drug on the CN polymer was influenced by the time, pH, concentration and temperature, while the ionic strength did not affect the drug adsorption by the polymer. Moreover, this study showed that cellulose modified with ethylenediamine is more efficient for removal of AMI from aqueous media than pure cellulose. This confirms that the incorporation of the nitrogenous groups on the surface of the cellulose improves the adsorbent characteristics of the cellulose. Therefore, it is proven that the CN polymer is effective both in the AMI adsorption, and can thus be used as a support for adsorption of other drugs similar to AMI.

## Figures and Tables

**Table 1:** Percentage of carbon and nitrogen from the polymer and the respective numbers of moles from pure and modified (CN) polymer.

Element	Pristine Microcrystalline Cellulose		CN Polymer	
	%	mmol·g^−1^	%	mmol·g^−1^
C	41.57 ± 0.15	34.64 ± 0.12	41.54 ± 0.02	34.62 ± 0.01
N	-	-	1.10 ± 0.01	0.78 ± 0.02

**Table 2:** Kinetic parameters obtained through the equation of pseudo-first order and pseudo-second order for the drug adsorption by the CN polymer at 298 K, and natural pH.

Model	Parameters	CN Polymer
Pseudo-first Order	*K*_1_	0.0101 (min^−1^)
	*q_e(cal)_*	58.7742 (mg·g^−1^)
	*R*^2^	0.9732
Pseudo-second Order	*K*_2_	0.0002 (g·mg^−1^·min^−1^)
	*q_e(cal)_*	72.4653 (mg·g^−1^)
	*R*^2^	0.9837

**Table 3:** Parameters obtained from Langmuir, Temkin and Freundlich isotherm models to AMI adsorption by CN polymer at 298 K, 308 K and 318 K, pH 7 and saturation time of 300 min.

Model	Parameters	CN Polymer
T = 298 K		
Langmuir	*q_max_*	273.2882 (mg·g^−1^)
	*K_L_*	0.0004 (L·mg^−1^)
	*R*^2^	0.9538
	*R_L_*	0.7180
Freundlich	*n_f_*	1.2637
	*K_f_*	0.3019 (L·g^−1^)
	*R*^2^	0.9433
Temkin	*n_T_*	13.7418
	*K_T_*	0.0060 (L·g^−1^)
	*R*^2^	0.9456
T = 308 K		
Langmuir	*q_max_*	234.5308 (mg·g^−1^)
	*K_L_*	0.0006 (L·mg^−1^)
	*R*^2^	0.9582
	*R_L_*	0.6143
Freundlich	*n_f_*	1.4190
	*K_f_*	0.6517 (L·g^−1^)
	*R*^2^	0.9387
Temkin	*n_T_*	18.1816
	*K_T_*	0.0065 (L·g^−1^)
	*R*^2^	0.9652
T = 318 K		
Langmuir	*q_max_*	311.7546 (mg·g^−1^)
	*K_L_*	0.0008 (L·mg^−1^)
	*R*^2^	0.5329
	*R_L_*	0.9627
Freundlich	*n_f_*	1.5923
	*K_f_*	1.2238 (L·g^−1^)
	*R*^2^	0.9371
Temkin	*n_T_*	17.6408
	*K_T_*	0.0074 (L·g^−1^)
	*R*^2^	0.9734

**Table 4:** Thermodynamic parameters for AMI adsorption by CN polymer.

Polymer	Δ*H*° (kJ·mol^−1^)	Δ*S*° (J·mol^−1^·K^−1^)	Δ*G*° (kJ·mol^−1^)		
			298 K	308 K	318 K
CN	7.7094	4.4285	6.3897	6.3454	6.3011
